# Frequent L1 retrotranspositions originating from *TTC28* in colorectal cancer

**DOI:** 10.18632/oncotarget.1781

**Published:** 2014-02-14

**Authors:** Esa Pitkänen, Tatiana Cajuso, Riku Katainen, Eevi Kaasinen, Niko Välimäki, Kimmo Palin, Jussi Taipale, Lauri A. Aaltonen, Outi Kilpivaara

**Affiliations:** ^1^ Genome-Scale Biology Research Program, Research Programs Unit, University of Helsinki, Helsinki, Finland; ^2^ Department of Medical Genetics, University of Helsinki, Helsinki, Finland; ^3^ Science for Life Center, Department of Biosciences and Nutrition, Karolinska Institutet, Stockholm, Sweden

**Keywords:** colorectal cancer, genome, sequencing, retrotransposon, L1

## Abstract

L1 element retrotranspositions have been found to alter expression of genes neighboring the insertion sites, potentially involving them in tumorigenesis and tumor progression. In colorectal cancer (CRC), L1 insertions have been found to target genes with a role in tumorigenesis. Structural changes such as L1 insertions are identifiable by whole genome sequencing (WGS). In this study, we observed frequent somatic L1 retrotranspositions originating from *TTC28* using deep coverage WGS data from 92 CRC tumor-normal sample pairs. In two cases the event had targeted *NOVA1* gene (*p*=0.025). In addition, a germline retrotransposition event from *TTC28* to *GABRA4* was found to be a common polymorphism in the Finnish population. Thus while some events may be tumorigenic, others are likely to be neutral. Our data contradict a recent study where a similar signal in *TTC28* was interpreted as a common inactivating translocation. While much work remains to be performed to understand the biological significance of retrotranspositions in cancer, accurate identification of these events is a prerequisite for success.

## INTRODUCTION

The role of transposable elements (TEs) in tumorigenesis and tumor progression is, despite active research [1-4], still largely unknown. L1 elements (Large Interspersed Element-1, or LINE-1) are transposable elements that amplify in the genome by retrotransposition [2]. Retrotransposition takes place via an RNA intermediate, leaving the origin intact. L1 sequences are very common, comprising ~17% of human genome [5]. However, only about 100 L1 elements are full-length (~6 kb) [6, 7], containing a promoter and two open reading frames (ORF1, ORF2), and thus capable of retrotransposition. These open reading frames encode an endonuclease and a reverse transcriptase, which are necessary to copy and paste the L1 sequence elsewhere in the genome. In somatic cells, activity of L1 elements is repressed by hypermethylation and post-transcriptional mechanisms [8, 2]. In cancer cells, however, hypomethylation is a common early event that allows L1 retrotransposition activity via loss of promoter methylation [8-11].

L1 retrotranspositions have been reported in several tumor types including colorectal cancer [1]. Colorectal cancers (CRCs), like most other solid tumors, display a variety of chromosomal changes such as deletions, inversions, translocations, amplifications, and other genetic abnormalities in addition to point mutations [12, 13]. The majority of such changes are passengers and a result of stochastic events and genetic instability observed in tumors, but a number of changes also contribute tumorigenesis. L1 retrotransposition is a structural change that may modify expression of the targeted gene. Although insertions of L1 elements are often 5' truncated, insertion of L1 sequence into an intron can for example truncate a transcript [14] or provide promoter for a novel transcript [15]. Furthermore, ORF2 endonuclease activity has been associated with aggressive prostate cancer phenotype [16] and excessive DNA double strand breaks [17]. In CRC, L1 retrotranspositions have been found to target genes including *ODZ3*, *ROBO2*, *PTPRM*, *PCM1*, and *CDH11* that have a role in tumorigenesis [4].

Whole genome sequencing (WGS) is a state-of-the-art method for analyzing structural variations in the genome [12] and is greatly contributing to our understanding of cancer genomes. In this work, we focused on a specific locus - *TTC28* intron 1 - that was recently reported to be the site for the most frequent structural change in CRC (~22% of cases; [18]). The change was interpreted in the study to be an inactivating translocation, thus depicting *TTC28* as a prime candidate for a new key CRC gene. We here challenge this interpretation based on investigation of our WGS data obtained from 92 paired CRC and normal tissue samples. We characterize a strikingly frequent somatic L1 retrotransposition originating from the first intron of *TTC28*. We find one of such retrotransposition to be a common polymorphism in the Finnish population.

## RESULTS

### Aberrant WGS signal in the first intron of *TTC28* stems from an L1 retrotransposition

**Figure 1:**
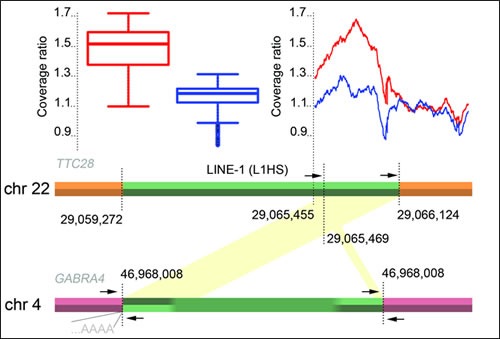
A schematic picture of the *TTC28-GABRA4* retrotransposition Primer locations (arrows), and relative mean coverage change in tumors are depicted: Mean coverages in cases with and without breakpoint signals were compared with the mean coverage of whole chromosome 22 across all tumors. Box plot illustrates the coverage ratios for the region 22:29,065,455-29,066,124 (GRCh37). Mean values for cases with a consistent discordant paired-end signal of at least three distally mapped mates are shown in red (n=54), the remaining cases in blue (n=38). Green region shows the originating L1 element in *TTC28* and the inserted sequence in *GABRA4*. Mapping of discordant read-pairs are shown in yellow. Location of the L1 poly-A insertion is also shown.

We initially identified an aberrant signal in 19 out of 92 (21%) WGS in the first intron of *TTC28* (chr 22: 29,065,455-29,066,124, GRCh37; Figure 1), where paired-end mates were mapped discordantly but consistently to a chromosome different from chr 22. This signal matched the location and frequency of the signal reported in [18], where it was interpreted as a translocation. On a closer visual inspection of mapped sequence data, however, we interpreted the signal to stem from a retrotransposition of an L1 element (L1Base id 129, [19]; dbRIP id 2000144, [20]) in the first intron of *TTC28*, instead of a translocation. The visual inspection also yielded additional cases with the retrotransposition signal that were not detected initially, described in detail below.

### L1 retrotranspositions originating from *TTC28* are frequent in CRC

**Figure 2:**
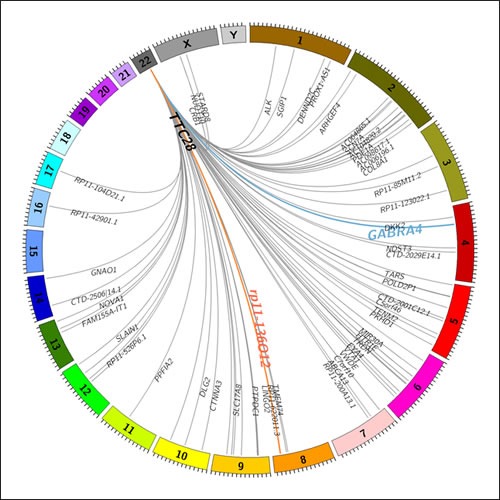
A Circos plot showing identified transposition events originating from *TTC28* locus in 92 CRC cases Germline transpositions to *GABRA4* and *rp11-136O12* loci are shown in blue and red, respectively. Somatic events are shown in grey. Insertion targets within genes are denoted by gene name.

A total of 83 somatic L1 insertions originating from a specific L1 element residing in the first intron of *TTC28* were observed in WGS data of 52 out of 92 (57%) CRC cases. Deep sequencing coverage (>40x) facilitated identification of multiple retrotransposition events in some of the cases. A total of 17 cases with two separate retrotransposition events were identified. In addition, we observed four cases that harbored three events, and two cases with four events. Insertion sites are illustrated in Figure 2 and reported in Supplementary Table 1. In 51 out of 83 retrotranspositions (61%), the insertion target was within an intron of a gene, implying significant intron preference (デ^2^=54.2, df=1, *p*=1.81e-13), compatible with previous literature on retrotranspositions [5]. All insertions which occurred within a gene hit a unique target except for two insertions targeting *neuro-oncological ventral antigen 1 (NOVA1)* (*p*=0.025).

### Sanger sequencing of somatic retrotransposition events

We successfully validated three somatic L1 insertions by Sanger sequencing. Insertion sites of the validated somatic events were in introns of *SGIP1*, *NOVA1* and *ARHGEF4*. In each case, we identified two PCR products of varying length: one matching the expected wild type allele size and a larger one representing the allele containing also the L1 insertion. In two cases (*SGIP1* and *NOVA1)*, we were able to see sequence corresponding to the truncated 5' end of the L1 element. In *SGIP1* case, we observed 449 bp of L1 sequence originating from 5484 bp downstream from the start of ORF1. Similarly in *NOVA1*, 246 bp of inserted L1 sequence originating from 5472 bp downstream from ORF1 start was identified. For the *ARHGEF4* case, we observed 235 bp of sequence from 6162 bp downstream from ORF1. Finally, in *SGIP1 and ARHGEF4* cases, we also observed a poly(A) tail at the 3' end.

### L1 retrotransposition events involving *TTC28* are also present in germline

In addition to the somatic retrotranspositions, two germline L1 retrotranspositions originating from *TTC28* were observed in WGS data; at *GABRA4* and *rp11-136O12* (Figure 2)*.* The retrotransposition from *TTC28* to *GABRA4* (Figure 1) was confirmed by Sanger sequencing and found to be present in 4/92 (4.3%) WGS sequences from CRC patients, in 3/90 (3.3%) additional CRC patients not used for WGS and in 9/90 (10%) anonymous Finnish blood donors, indicating that the aberration is a common polymorphism in Finns. An inversion of the 3' end of the original L1 sequence was seen in the inserted site (Figure 1).

The four WGS cases sharing the germline retrotransposition at *GABRA4* (chr 4) were found to share significantly longer haplotypes around the insertion locus than other cases, indicating a shared founder haplotype (t=2.6, *p*=0.046, 95% CI [74353,6150003]). As expected, evidence for shared haplotypes at *TTC28* (chr 22) were not observed in cases sharing the *TTC28*-*GABRA4*-retrotransposition (t=-0.27, *p*=0.79). The 3/92 (3.3%) WGS cases with the germline L1 insertion at *rp11-136O12* (chr 8) were found not to have a significantly longer haplotype (t=-0.27, *p*=0.81), suggesting more ancient or independent origins.

### Gain of L1 sequence from the *TTC28* first intron in retrotransposition cases

Finally, we examined the copy number changes around the 3' end of *TTC28* L1 element (Figure 1), and found that these data too were compatible with L1 retrotransposition rather than translocation, in both germline and tumors displaying the aberrant WGS signal. Since the sequence in the 3' end of the L1 element in *TTC28* allowed unique mapping of paired-end reads, copy number analysis could be performed with WGS data. By comparing the tumor mean read coverage in the L1 3' end region to the whole chromosome 22, we discovered that in tumors with *TTC28 “*breakpoints” the relative coverage was significantly increased compared to cases where no retrotransposition signal was observed (t=49, *p*<2.2x10^−16^, 95% CI [0.32, 0.34]; Figure 1). This result indicates gain of L1 sequence (Figure 1) in cases displaying the retrotransposition signal, suggesting that this sequence in chromosome 22 was present in more than two copies.

## DISCUSSION

Transposable elements, such as L1, and their role in cancer are under active study [1, 2, 3]. We observed strikingly frequent L1 retrotranspositions in 57% of colorectal cancers originating from an L1 element in *TTC28*. In total, 83 somatic and 7 germline retrotranspositions that had originated from the element were observed. This remarkably active retrotransposition was the most frequent structural change detected in our WGS data consisting of 92 tumor-normal pairs. The retrotransposed sequence, belonging to the L1 subfamily TA-1nd, has earlier been found to be one of the most active L1 elements in a cultured cell retrotransposition assay [6].

Identification of retrotranspositions by WGS is often difficult due to the large number of almost identical copies of retroelements [1]. Detection of the particular retrotranspositions studied here and their origin in *TTC28* was possible due to the distinct 3' UTR of the element, which allowed identification of the source locus by short-read mapping*.* In many cases, however, accurate identification of retrotransposition source locus is not feasible using only short-read data. Besides shedding light on the activity of this particular transposable element in cancer, our result highlights the importance of careful analysis of WGS data and robust validation of candidate aberrations. Reliable detection of structural variants, such as events involving transposable element insertions, requires sufficient sequencing coverage. Here we were able to observe multiple retrotransposition targets per sample stemming from the same *TTC28* locus due to sufficiently deep coverage.

The frequent breakpoint signals in *TTC28* (22:29,065,671-29,066,377) observed by TCGA [18] and those in our study reside in the same narrow region and are very likely to reflect the same phenomenon. Our interpretation contradicts that presented in the TCGA study where *TTC28* was depicted as a frequent target for inactivating translocations [18]. The interpretation by TCGA was based on WGS calls, as well as validation of some of the breakpoints by PCR and Sanger sequencing. In our study, we managed to validate both junction breakpoints related to the retrotransposition. Such validation is required to identify this particular structural change as a retrotransposition instead of a translocation involving only one breakpoint at the originating locus. Overall, we strongly suggest that the changes interpreted by the TCGA as inactivating translocations involving *TTC28* are in fact L1 retrotranspositions, based on the evidence presented here.

Retrotranspositions may have oncogenic effects [21], or they can be neutral depending on their insertion sites. In our data, all insertions hit separate targets, except for two insertions targeting *NOVA1*. Given the large number of potential insertion sites, even two hits to a single gene was somewhat unexpected (*p*=0.025) and might reflect selective value. *NOVA1* has been identified as a splicing factor playing a role in neuronal splicing program, but it is also expressed in fibroblasts [22]. Other splicing factors such as *SRSF6* have been associated with colorectal cancer [23]. The possible role of *NOVA*1 in CRC remains to be studied. We also found that *TTC28-GABRA4* retrotransposition is a common polymorphism in the Finnish population. Thus this particular event is not likely to be oncogenic.

To summarize, our study sheds light on the nature of *TTC28* aberrations in CRC, as well as provides a valuable lesson in interpretation of WGS data. The *TTC28* events that we observed are frequent, and some may be involved in tumorigenesis while others are likely to be neutral. Much work remains to be done to unravel the biological consequences of retrotranspositions in cancer. Accurate identification of these events is a prerequisite for success.

## METHODS

### Study samples

In total, 92 familial CRC cases from 89 families (tumor and normal DNA) fulfilling the following criteria were included in this study: (i) at least one CRC case in a first degree relative, (ii) negative for any known high penetrance CRC mutation, and (iii) availability of sufficient amount of DNA, and (iv) microsatellite stable tumors. Seventy-nine of these 92 cases, and the additional 90 cases, used in the validation are part of a previously described population-based collection of Finnish CRC cases [24, 25]. The rest of the CRC cases (n=13) are part of an unpublished sample series from two Finnish hospitals. Finnish blood donor DNA samples (n=90) obtained from the Finnish Red Cross Blood Transfusion Service were used as controls.

The study has been reviewed and approved by the Ethics Committee of the Hospital district of Helsinki and Uusimaa (HUS). Signed informed consent or authorization from the National Supervisory Authority for Welfare and Health has been obtained for all the study participants.

### Whole-genome sequencing of 92 CRC tumor and normal DNA samples

Whole-genome sequencing was performed on the Illumina HiSeq 2000 platform with paired-end reads of length 100 bp. Each normal and tumor DNA sample was sequenced to at least 40x median coverage. Sequencing data quality was evaluated with FastQC (www.bioinformatics.babraham.ac.uk/projects/fastqc). Paired-end sequencing data was aligned using BWA 0.6.2 [26] against the 1000 Genomes Project Phase 2 reference assembly, which is derived from the GRCh37 reference [27]. Default BWA parameters were used, except for -n 0.06, -q 5 for bwa aln and -a 800 for bwa sampe. PCR duplicates were removed with samtools [28]. Local realignment was performed by GATK around known indel sites in 1000 Genomes and Mills gold standard sets, and 1000 Genomes Phase 1 indels, in addition to preliminary indel calls created using GATK UnifiedGenotyper [29]. Base quality scores were recalibrated with GATK.

### Identification of somatic structural changes

Somatic structural aberrations were identified in WGS with DELLY [30] and custom scripts. DELLY is a computational method to detect deletions, tandem duplications, inversions and translocations in whole-genome paired-end sequencing data using paired-end and split-read signatures. Structural changes with respect to the reference genome were identified independently in tumor and normal samples. In tumors, a minimum mapping quality of 20 and at least five supporting reads were required to make a call. To make normal calls for subsequent somatic filtering, mapping quality threshold was not used and only two supporting reads were required, resulting in a highly sensitive call set. To identify somatic retrotransposition events, the following filtering approach was adopted. Breakpoints of translocation calls in each normal sample were first flanked by 500 bp in both directions. Any translocation called in a tumor sample where a breakpoint of the translocation was in the combined flanked regions of respective normal sample was removed. This process yielded a set of somatic translocation calls for each tumor-normal sample pair. Each translocation call was annotated with genes containing either of translocation breakpoints. A similar filtering approach to somatic translocation identification was followed in our previous whole-genome study [31].

### Calling retrotransposition events at *TTC28* in WGS data

The most frequently involved gene in DELLY translocation calls was *TTC28* on chromosome 22. Breakpoints of translocation calls involving *TTC28* were further investigated by manual inspection of aligned paired-end sequences using RikuRator genome analysis software (manuscript under preparation). Here it was observed that the presumed translocation calls corresponded to insertions of sequence originating from the *TTC28* locus elsewhere in the genome, instead of a translocation. Whole-genome sequences of all tumor and normal samples at the breakpoint position were visually inspected in RikuRator. An L1 insertion to a specific locus was called when at least three paired-end reads supported the insertion. This inspection also revealed the germline insertions that were initially removed by the above somatic filtering.

### Copy number analysis

Copy number changes around the 3' end of the L1 element in *TTC28* were examined by first calculating the sequencing coverage in tumors around the 3' end of the L1 element (chromosome 22:29,065,455-29,066,124, GRCh37; Figure 1). This region of 669 bp was chosen based on the *GABRA4* germline case, where it is copied in the retrotransposition (Figure 1). In particular, the region is unique to the reference genome (GRCh37), allowing discordant read and copy number analysis of the specific locus. For each tumor sample, the ratio of mean coverages in the region and chromosome 22 was computed. A t-test was employed to assess whether there is a significant difference in coverage ratios between cases with either a germline or somatic retrotransposition, and cases with no detected retrotransposition.

### Genotyping and haplotype analysis

Each sample was genotyped on the Illumina HumanOmni2.5-8 BeadChip platform containing 2,379,855 markers. SNP calling was performed using Illumina GenomeStudio. Shared haplotypes around L1 breakpoints in germline cases were identified in genotyping data by extending a candidate haplotype from a given position to both 5' and 3' directions. Extension was terminated when a pair of opposing homozygotes was found such that the sites were separated by less than 2000 bp. Prior to analysis, the three first-degree relatives were removed, leaving 89 cases to be studied. A t-test was used to assess whether the haplotypes at *GABRA4* and *TTC28* loci found in this manner were significantly longer in cases sharing a *GABRA4*-targeting retrotransposition than in other cases. The other germline L1 insertion target, *rp11-136O12*, was tested identically to *GABRA4.*

### Insertion site validation by Sanger sequencing

One germline L1 insertion site candidate (*GABRA4*) was validated by Sanger sequencing in four samples. Primer pairs comprising each insertion junction were designed using Primer3Plus ([32]; http://www.bioinformatics.nl/cgi-bin/primer3plus/primer3plus.cgi/); primer sequences and PCR conditions are available upon request. Fragments were amplified with AmpliTaqGoldVR (Applied Biosystems, Foster City, CA) and the PCR products were purified using the ExoSAP-IT PCR purification kit (USB, Cleveland, OH). Sequencing reactions were performed using the Big Dye Terminator v.3.1 kit (Applied Biosystems) and electrophoresis was run on 3730xl DNA Analyzer (Applied Biosystems) at FIMM Genome and Technology Centre, Finland. The sequence graphs were manually analyzed using FinchTV v.1.4 (Geospiza, Seattle, WA).

Additional three somatic L1 insertion candidates (*SGIP1*, *NOVA1*, and *ARHGEF4*) were successfully validated by Sanger sequencing. Primers flanking the insertion breakpoints were designed, as previously described. The PCR was performed using Expand Long Template PCR system (Roche, Basel, Switzerland). The PCR products were run in standard low-melting agarose gel. DNA band corresponding to the allele with the L1 insertion, was extracted from the agarose gel using QIAquick Gel extraction Kit (Qiagen, Hilden, Germany). The sequencing reaction and the analysis of the sequences were performed as previously described.

### Permutation testing of NOVA1 significance

Significance of *NOVA1* somatic hit recurrence was assessed with a permutation test. In each permutation, the target of each of 83 somatic events was randomly reassigned to either a gene (61% chance) or intergenic region (59%). In case of an event targeting a gene, the target gene was uniformly selected from 51573 genes, including protein-coding genes, short and long non-coding RNAs and pseudogenes (Ensembl 71.37). A total of 1000000 permutations were performed. An empirical *p*-value was derived as the fraction of permutations where any gene was hit two or more times.

## Supplementary Figure


